# Neuroimmune modulatory effects of the NLRP3 inflammasome: the role of sex and living environment in brain affecting conditions

**DOI:** 10.3389/fimmu.2025.1711656

**Published:** 2025-11-20

**Authors:** Miriam Ciani, Giovanna Rigillo, Beatrice Bertarini, Cristina Benatti, Silvia Alboni, Fabio Tascedda

**Affiliations:** 1 Department of Life Sciences, University of Modena and Reggio Emilia, Modena, Italy; 2 Department of Biomedical, Metabolic and Neural Sciences, University of Modena and Reggio Emilia, Modena, Italy; 3 Consorzio Interuniversitario Biotecnologie (CIB), Trieste, Italy

**Keywords:** NLRP3 inflammasome, biological sex, central nervous system, social isolation, enriched environment, exposome, pharmacology

## Abstract

The NLRP3 inflammasome is a master regulator of neuroinflammation, linking systemic perturbations to brain dysfunction and thereby influencing overall brain health. Its sensitivity to biological sex and environmental factors suggests that NLRP3 may act both as a contributor to sex-dependent disease mechanisms and a modifiable therapeutic target for pharmacological and non-pharmacological interventions. In this mini-review, we summarize emerging evidence on sex-specific differences in NLRP3 signaling that may contribute to disparities between males and females in disease incidence, symptomatology, and treatment response. Neuroinflammation-driven disorders, including atherosclerosis, neuropathic pain, substance use, and stress-related syndromes, show how sex influences NLRP3 inflammasome expression and activity with downstream effects on cognition and behavior. We also examine the modulatory influence of environmental factors, with emphasis on social behavior and environmental enrichment, as determinants of NLRP3 dynamics relevant to neurocognitive function and brain health. Overall, the findings suggest that NLRP3 acts as a central hub integrating sex and environmental influences, with broad implications for personalized interventions in brain-related disorders.

## Graphical Abstract

The NLRP3 inflammasome emerges as a central hub at the intersection of biological sex and exposome influences, integrating genetic, hormonal, and lifestyle-related inputs. Its dysregulation contributes to multiple brain-related conditions, including vascular disorders, neuropathic pain, aging, stress responses, and substance abuse. Understanding these modulatory axes highlights opportunities for precision NLRP3-targeted therapies tailored to sex-specific and environmental determinants.

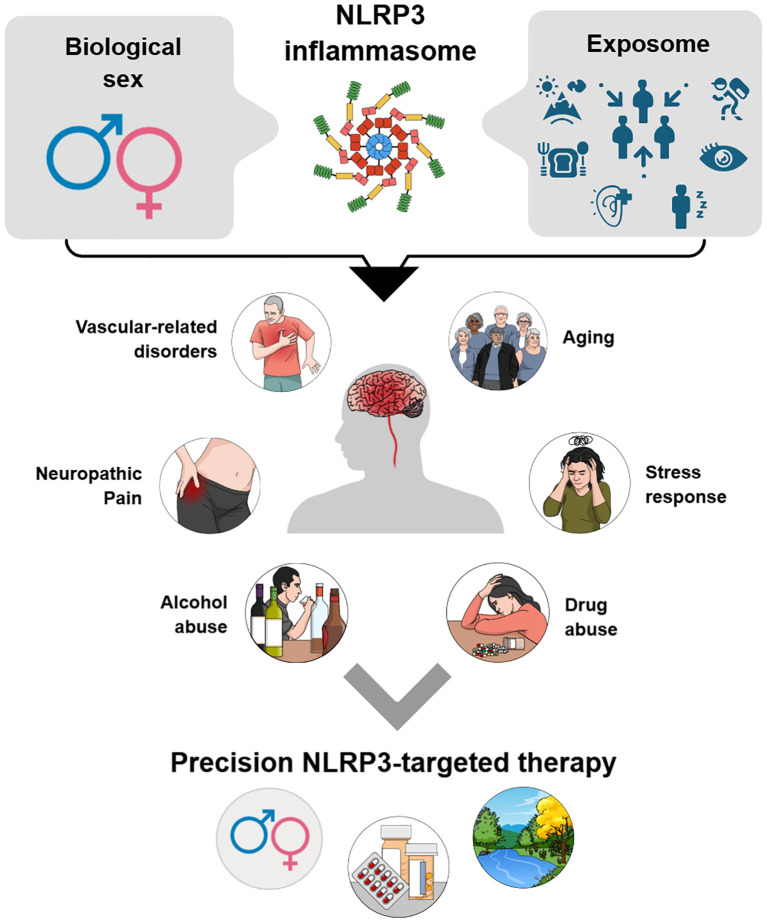

## 1 Introduction

The inflammasome family comprises cellular multiprotein complexes that detect stress and danger signals, supporting host defense and damage resolution. Key platforms include NLRP1, NLRP3, NLRC4, AIM2, and Pyrin, with NLRP3 being the best-characterized regulator of early neuroinflammatory responses (1, 2). Its activation occurs in two phases: (i) priming, defined by NF-κB-dependent transcriptional upregulation of NLRP3, pro-IL-1β, and pro-IL-18; and (ii) activation triggered by stressors such as mitochondrial dysfunction, ROS production, potassium efflux, and lysosomal rupture, leading to assembly with ASC and pro-caspase-1 (3). This cascade culminates in caspase-1 activation, GSDMD-mediated pyroptosis, and maturation of IL-1β and IL-18 (4, 5). Although protective, dysregulation fosters chronic inflammation with profound systemic consequences, including impaired brain function. NLRP3 overactivation contributes to neurodegeneration across multiple conditions, such as depression, Alzheimer’s disease (AD), ischemic injury, and metabolic-associated cognitive decline, making it a major therapeutic target (6–9).

Emerging data highlight sex-based differences in NLRP3 expression, regulation, and function, shaping disease risk, patient outcomes, and treatment efficacy, and underscoring the importance of including sex as a biological variable in neuroimmunology and inflammasome-targeted therapies (10–12). NLRP3 is also highly sensitive to environmental inputs, including sensory stimulation, physical activity, and psychosocial experiences, which modulate its activity and impact brain development, neuroplasticity and function (13–15). Thus, NLRP3 emerges as a modifiable determinant of neurobehavioral health and disease vulnerability.

Building on this, this mini-review integrates current evidence on emerging sex-related differences in NLRP3 inflammasome across conditions involving cognitive and behavioral impairments. In parallel, within the broader context of environmental influences, we examine two critical dimensions of social and environmental exposure, social isolation and environmental enrichment, and their effects on the NLRP3 pathway with downstream implications for cognition, behavior, and brain health.

## 2 Methods

The literature search was performed in PubMed, Web of Science, Scopus, and Google Scholar, restricted to English publications and updated to August 15, 2025. Keywords included “*NLRP3 inflammasome*,” “*sex differences* OR *male* OR *female*,” “*enriched environment*,” “*social isolation*,” “*exercise* OR *physical activity*,” “*central nervous system*,” “*cognition*”. Eligible studies were *in vivo* models assessing sex differences and/or environmental influences on NLRP3 activity in CNS-related conditions. Exclusion criteria were *in vitro*-only studies, non-English reports, narrative reviews, or works without NLRP3 measures. In total, 25 studies, mostly preclinical, were included, addressing how sex and environmental factors, conceptualized as somatosensory-rich contexts and social behaviors, modulate NLRP3 signaling in CNS pathophysiology.

## 3 Sex-specific modulation of the NLRP3 inflammasome in systemic disorders affecting brain health

Sex differences in immune-inflammatory responses are increasingly recognized for their impact on health and disease. Biological sex shapes immune activity at peripheral and central levels across the lifespan through interaction among hormones, chromosomal factors, and environmental influences (16–19). Males and females differ in the magnitude and quality of inflammatory responses, including immune cell activation, signaling pathways, and downstream processes (20). These variations influence disease susceptibility, progression, and therapeutic outcomes (21–23). The NLRP3 inflammasome is emerging as a pathway modulated by sex hormones, which exert context-dependent effects and contribute to vulnerability or resilience in brain disorders (23–25). Sex-related differences in NLRP3 signaling are reported both at baseline and under inflammatory challenges, varying across tissues, developmental stages, and disease models (26, 27). The following section examines CNS related conditions where NLRP3 is crucial, focusing on *in vivo* evidence of sex-dependent differences in its expression and activity (**Table 1**).

**Table 1:** Experimental evidence of sex differences in NLRP3 inflammasome expression/activation across systemic conditions with neurological impact.

Paradigm	Experimental model	Effect on NLRP3 inflammasome	Mechanism involved	Functional outcome
Vascular-related disorders	C57BL/6J Ldlr^-/-^ mice, female Nlrp3^-/-^ chimera and Nlrp3^-/-^ mice; ovariectomized and castrated conditions	Male: Nlrp3^-/-^ no protection. Female: Nlrp3^-/^: ↓ lesions, ↓ lipid content	Estrogen promotes, while testosterone blocks, NLRP3 inflammasome-mediated inflammation	Immunological sex differences in atherosclerosis environment (12)
Vascular-related disorders	C57BL/6J Ldlr^-/-^ male and female mice	↑ NLRP3 in female aortas	Macrophage-driven NLRP3–IL-1β inflammatory activation	Sex differences in the atherosclerotic immune landscape (28)
Postoperative Pain	C57BL/6J mice: neuron-specific NLRP3 cKO and WT mice; skin and muscle plantar incision	↑ NLRP3, ↑ IL-1β in males	Microglia-neuron IL-1β signaling mediates pain hypersensitivity	Faster recovery in males lacking NLRP3; minimal effect in females (29)
Neuropathic pain	Sprague Dawley rats: Chronic Constriction Injury (CCI)	↑ NLRP3, AIM2 in females; NLRP1 ↑ in males; IL-1β mRNA ↑ in females	Microglia/astrocyte-specific inflammasome expression	IL-1r antagonist reverses established pain in both sexes; differential inflammasome gene expression (30)
Chronic Pain	C57BL/6J mice; λ-carrageenan- induced hyperalgesic priming/PGE2 injection/monoiodoacetate (MIA)-induced osteoarthritis	↑ NLRP3 in DRG; ↑ oxidative stress; MCC950 prevents transition to chronic pain	Mitochondrial dysfunction activates NLRP3 in sensory neurons	Persistent pain prevented by NLRP3 inhibition; longer lasting in males (31)
Prenatal Alcohol Exposure (PAE)/allodynia	C57BL/6J mice: PAE + sciatic nerve injury + morphine	↑ NLRP3, IL-1β, HMGB1, caspase-1 activity; reversed by MCC950	TLR4 → NLRP3 inflammasome → IL-1β cascade; enhanced glial activation	Prolonged morphine-induced allodynia; minor sex-specific effects (32)
Acute Ethanol Exposure	Wistar rats: *ex vivo* BLA slices + *in vivo* EtOH injection	Males: NLRP3 activation → ↑ GABA release from interneurons Females: no effect	TLR4 → NLRP3 → IL-1R pathway	Males: reduced anxiety-like behavior Females: no behavioral effect (33)
Alcohol use Disorder	C57BL/6J mice: two-bottle choice alcohol test + MCC950, VX765, IL-1ra	Inhibition of NLRP3 inflammasome, caspase-1, IL-1 receptor	Inhibition of different steps in inflammasome signaling	Reduced alcohol intake and preference in females (34)
Psycoactive drug use	Heterogeneous Stock (HS) rats: cocaine self-administration	↑ NLRP3 in males; ↑ pro- and active caspase-1, IL-1β, and ASC dimers in females. ↑ NF-κB in both sex	Sex- and region-specific inflammasome regulation: males show priming (striatum), females show activation (hippocampus); possible divergence in microglial vs neuronal engagement	Cocaine can exert region- and sex-specific differences in neuroimmune signaling in the brain is possibly linked to different moods and cognitive vulnerability (35)
Stress	C57BL/6 mice treatment with LPS	Males: ↓ NLRP3, IL-1β, IGF-1, ↑ TREM2 Females: ↑ NLRP3, IL-1β	Sex-specific microglial activation	Males: depressive-like behavior Females: increased inflammation without behavioral change (36)
Stress	Sprague-Dawley rats; single episode of Acute Immobilization Stress (AIS)	Males: ↑ NLRP3 post-stress Females: higher baseline NLRP3 but no increase post-stress	Sexually dimorphic regulation of peripheral cytokines and hippocampal NLRP3 expression	Males: robust pro-inflammatory response Females: blunted response (37)
Neonatal stress	Sprague-Dawley rats (PND7): treatment with LPS and sevoflurane	No significant NLRP3 activation; ↑ NLRP1, caspase-1, IL-1β, IL-18	caspase-1- mediated neuroinflammation	Males: learning/memory deficits Females: anxiety-like behavior (38)
Aging	C57BL/6J mice at 3 and 18 months old	↑ IL-1β, ASC, caspase-1 in female cortex	Sex differences in cortical inflammaging	Sex differences in the expression of inflammatory targets to prevent the development of neurodegenerative disease (39)
Aging	C57BL/6J mice: (APP/PS1+/-) AD-like model	↑ NLRP1, caspase-1, and IL-1β in female cortex	Activation of NLRP1 inflammasome contributed to Aβ aggregation and neuroinflammation in the CNS	Sex difference of NLRP1 signals in the brains may contribute to the development of AD (40)

AD, Alzheimer’s disease; AIS, acute immobilization stress; ASC, apoptosis-associated speck-like protein containing a CARD; BLA, basolateral amygdala; CCI, chronic constriction injury; CSF, cerebrospinal fluid; DRG, dorsal root ganglia; GSDMD, gasdermin-D; IL-1β/IL-18, interleukin-1β/-18; LPS, lipopolysaccharide; MCC950/CRID3, direct NLRP3 inhibitor; mtDAMPs, mitochondrial danger-associated molecular patterns; NEK7, NIMA-related kinase-7; NF-κB, nuclear factor kappa-B; PAG, periaqueductal gray; PM2.5, fine particulate matter; PND, postnatal day; ROS, reactive oxygen species; TLR4, Toll-like receptor-4.

### 3.1 Vascular-related disorders

Vascular dysfunction, manifesting endothelial impairment, altered cerebrovascular reactivity, or vessel damage, is a hallmark of multiple CNS affecting disorders by promoting neuroinflammation. Extensive evidence emphasizes the causal role of NLRP3 in driving endothelial dysfunction and atherogenesis (41–43). Targeting NLRP3 reduces vascular inflammation and delays CNS complications (44, 45). Differences in atherosclerosis between sexes may, in part, arise from varied activation of NLRP3 (12, 28) (**Table 1**). Chen et al. (2020) demonstrated that NLRP3 contributes to atherogenesis in a sex-dependent manner via estrogen-mediated regulation: their study demonstrated that ovariectomy condition in a valuable model of atherosclerosis, such as the low-density lipoprotein receptor gene knockout mice (Ldlr^-^/^-^), revealed that NLRP3 deficiency markedly reduced plaque formation in middle-aged females, lowering caspase-1 activation, IL-1β release, and immune cell infiltration. These protective effects were absent in castrated males, where testosterone depletion heightened reliance on NLRP3 activity (12).

A recent multi-modal immunophenotyping study showed that biological sex shapes immune cell composition in aged atherosclerotic plaques via NLRP3 signaling: aged Ldlr^-^/^-^ females exhibited stronger pro-inflammatory macrophage responses, whereas males displayed a distinct profile marked by greater CD8^+^ T cell and dendritic cell involvement (28). Together, these studies indicate that estrogen amplifies NLRP3-driven vascular inflammation, while testosterone exerts a protective, anti-inflammatory role in atherosclerotic progression.

### 3.2 Neuropathic pain

Chronic neuropathic pain, marked by maladaptive central sensitization, involves dysfunction of the prefrontal cortex, hippocampus, and amygdala (46, 47). A shared mechanism across chronic pain syndromes (e.g. diabetic neuropathy, fibromyalgia, and spinal cord injury) is the neuroimmune activation, largely mediated by microglial NLRP3 signaling. This promotes IL-1β/IL-18 releases and sustains pain through neuroinflammatory feedback loops (48, 49). Growing recognition exists for sex-based differences in pain perception with distinct cellular and molecular inflammasome dynamics (48–51) (**Table 1**). In a postoperative pain model, Cowie et al. (2019) showed that males relied on both neuronal and non-neuronal NLRP3 for IL-1β-mediated hypersensitivity, whereas females depended exclusively on neuronal NLRP3, making inhibition less effective (29). Using a chronic constriction injury (CCI) model of the sciatic nerve, Green-Fulgham et al. (2024) reported sex-specific inflammasome signaling in the spinal cord: female mice showed higher IL-1β, NLRP3, and AIM2 expression, whereas males exhibited increased levels of NLRP1. Notably, IL-1 receptor antagonist treatment reversed pain behaviors in both sexes, indicating shared therapeutic pathways despite divergent molecular mechanisms (30). In an osteoarthritis model, Ribeiro et al. (2023) found that neuronal NLRP3 involvement in dorsal root ganglia, driven by mitochondrial dysfunction, mediated the transition to chronic pain. Pharmacological inhibition of NLRP3 with the selective inhibitor MCC950 was effective in both sexes, but had longer-lasting effects in males, suggesting sex-dependent regulation (31). Additionally, prenatal alcohol exposure also exacerbated adult pain via NLRP3 hyperactivation, especially in male mice, accompanied by spinal glial reactivity and upregulation of HMGB1, a key amplifier of inflammasome activation. MCC950 treatment reversed these changes, indicating long-term sex-specific neuroimmune programming. Finally, spared nerve injury-induced pain in female mice caused stronger mechanical hypersensitivity and greater NLRP3 activation in the midbrain periaqueductal gray compared with males, further supporting sex-dimorphic inflammasome activity in central pain circuits (32).

### 3.3 Substance use disorders

Chronic substance use induces persistent neurobiological changes impairing behavior, motor function, emotion, and cognition (52). Individuals with substance use disorders face greater risk from comorbidities, drug interactions, and polypharmacy, which exacerbate neuroinflammation (53). The NLRP3 inflammasome is a pivotal mediator of addiction, activated by alcohol and other psychoactive agents in both brain and periphery (54, 55). Indeed, its inhibition has been demonstrated to reduce drug-seeking, withdrawal anxiety, relapses, and neurobehavioral symptoms, underscoring therapeutic potential (34, 56).

#### 3.3.1 Alcohol consumption

Alcohol abuse compromises brain immunity through NLRP3 inflammasome overactivation, driving caspase-1 activation, IL-1β release, pyroptosis, and impaired autophagy/mitophagy and mitochondrial function, which further sustain pathway activity (57–61). These mechanisms, prominent in the prefrontal cortex, hippocampus, amygdala, cerebellum, and cerebral cortex, underlie behavioral and cognitive deficits (62–65). Although microglial NLRP3 activation is central, neurons, astrocytes, and impaired microglia-neuron communication also contribute (60, 65). Notably, pharmacological NLRP3 inhibition reduces inflammation and improves behavioral outcomes in alcohol-related brain injury (66, 67).

Although early studies focused on male models, recent findings highlight sex-dependent differences in NLRP3 activation and alcohol responses (**Table 1**). Munshi et al. (2023) showed that acute alcohol exposure in adolescent and young adult rats activated NLRP3 in the basolateral amygdala only in males, inducing GABAergic inhibition and anxiety-like behaviors, while females remained unaffected (33). Similarly, Lowe et al. (2020) reported that inflammasome inhibition via MCC950, VX765 (caspase-1 inhibitor), or anakinra (IL-1 receptor antagonist) reduced alcohol intake in females, whereas in males only MCC950 and anakinra were effective, suggesting sex-specific differences in downstream inflammasome signaling (34). Together, these findings indicate that alcohol-related modulation of NLRP3 signaling is strongly sex-dependent. Males appeared more vulnerable to NLRP3-mediated neuroinflammatory and behavioral alterations, whereas females displayed differential sensitivity to inflammasome inhibition, suggesting distinct downstream signaling or compensatory immune mechanisms between sexes.

#### 3.3.2 Psychoactive drug consumption

Opioids, stimulants, and other psychoactive substances trigger NLRP3 inflammasome activation in central regions such as the dorsal raphe nucleus, hippocampus, piriform cortex, and amygdala, initiating neuroinflammation that impairs cognition, mood, and pain processing, also promoting neurodegeneration (55, 68–73). Oxidative stress, mitochondrial dysfunction, and impaired autophagy, exacerbate neural vulnerability (54). NLRP3 activation occurs in microglia, astrocytes, and neurons varying by drug and cell type. Pharmacological targeting of this pathway shows promise in reducing substance-induced neurotoxicity and behavioral alterations (69). Notably, Cheng et al. (2023) demonstrated that cocaine self-administration induced neuroinflammation through sex-specific mechanisms: in females, hippocampal NLRP3 activation occurred independently of CRF signaling, which regulates the NF-κB-NLRP3 pathway via the HPA axis; in males, by contrast, striatal NLRP3 expression was increased alongside CRF signaling (35) (**Table 1**).

### 3.4 Stress response

Stress is a physiological process by which the brain reacts to physical or psychological threats. Activation of the fight-or-flight system releases stress hormones and triggers systemic changes that, while adaptive short term, become maladaptive when chronic, particularly within the CNS, increasing vulnerability to neuropsychiatric and neurodegenerative disorders (74–76). The NLRP3 inflammasome links cellular stress to neuroinflammation and its sustained activation contributes to cognitive, emotional, and structural brain alterations (77–80). Biological sex further shapes stress responses, driving divergent inflammasome activation profiles (81, 82). Evidence indicates that NLRP3 expression and responsiveness vary with sex, as well as tissue, developmental stage, and disease context (**Table 1**).

In adulthood, systemic inflammatory stress differentially modulates NLRP3 across sexes. Alonaizan et al. (2025) reported that acute LPS-induced neuroinflammation engaged NLRP3 in both male and female mice, yet only males developed behavioral impairments, indicating a sex-specific vulnerability despite comparable microglial activation (36). Likewise, Sood et al. (2022) observed higher basal hippocampal NLRP3 levels in females, but stress-induced upregulation occurred only in males, suggesting heightened inflammasome sensitivity in the male brain (37). Sex differences are also evident during neurodevelopment. Useinovic et al. (2022) demonstrated that neonatal (postnatal day-PND7) exposure to LPS plus the anesthetic sevoflurane enhanced hippocampal NLRP3-mediated neuroinflammation and induced long-term behavioral changes. Males showed learning and memory deficits, whereas females exhibited increased anxiety-like behaviors, underscoring the interplay between early-life inflammation, pharmacological exposure, and sex-specific inflammasome signaling (38).

### 3.5 Aging

Aging is a major risk factor for neurodegenerative and neuropsychiatric disorders, largely through dysregulation of immune-inflammatory pathways in the CNS. While transient inflammation may be protective, aging is accompanied by chronic low-grade inflammation (“*inflammaging*”) that promotes neuronal vulnerability, glial dysfunction, and cognitive decline (83). The NLRP3 inflammasome is a key mediator, sensing age-related stressors and amplifying neuroinflammation via caspase-1 activation, IL-1β/IL-18 releases, and pyroptosis (84, 85). Evidence suggests that NLRP3 signaling increases with age, and it follows sex-specific trajectories across the lifespan, fostering shaping susceptibility and disease progression (**Table 1**).

Cyr et al. (2023) showed that aged female mice exhibit a markedly enhanced pro-inflammatory transcriptional profile in cortical and hippocampal regions, with higher expression of caspase-1 and IL-1β compared with males (39), although these changes may reflect a broader upregulation of inflammasome pathways beyond NLRP3 specifically. By contrast, male brains displayed relatively stable or even decreased inflammasome-related gene expression across aging. Similarly, Zhang et al. (2020) reported that in an AD-like mouse model, expression of NLRP1, caspase-1, and IL-1β was more elevated in females than in males, pointing to heightened inflammasome activation in the aging female brain (40).

## 4 Environmental modulation of the NLRP3 inflammasome in brain health and disease

The lifelong imprint of environmental exposures, defined as the *exposome*, is a major determinant of brain health and disease susceptibility (86, 87). It encompasses non-genetic factors, including diet, physical activity, sleep patterns, environmental pollutants, stress, infections, sensory stimuli, and the social milieu, all strongly shaping neuroimmune function and plasticity (88–91). Many influences converge on NLRP3 inflammasome modulation through priming (e.g., NF-κB transcription) and activation triggers (e.g., mitochondrial ROS, K^+^ efflux) (15, 92–95).

Diet and nutraceuticals regulate priming via the gut–microbiota–brain axis, mediated by short-chain fatty acids and microbial metabolites (96–102). Circadian disruption and sleep loss heighten inflammatory tone by enhancing microglial NLRP3 activity (103–105), while pollutants amplify oxidative stress and neuroinflammation, accelerating degeneration (106–109). Conversely, physical activity broadly suppresses NLRP3 signaling, supporting cognitive resilience (110, 111). Of note, greater lifelong exposure to positive social interactions, physical activity, sensory stimulation, and cognitive engagement, beyond standard conditions, has been identified as a powerful protective factor for brain and systemic health, thereby lowering the risk of inflammatory and neurodegenerative disorders.

Social and environmental influences, from social connection to sensory engagement, profoundly modulate brain health directly link inequalities to health disparities. Indeed, favorable and adverse conditions differentially shape NLRP3 activation and, in turn, cognitive and behavioral outcomes, with important implications for brain well-being and relevance to both neurodegenerative and psychiatric disorders (7, 112). Within this exposome-informed framework, we next summarize evidence of social isolation and environmental enrichment as key exposures that regulate NLRP3 activity *in vivo*, producing divergent effects on cognition and behavior (**Table 2**).

**Table 2:** Experimental evidence of living environment effects on NLRP3 inflammasome expression/activation across systemic conditions with neurological repercussions.

Paradigm	Experimental model	Effect on NLRP3 inflammasome	Mechanism involved	Functional outcome
Enriched Environment	Sprague-Dawley rats: MCAO/R	↓ NLRP1/NLRP3, caspase-1, GSDMD, IL-1β/IL-18	NF-κB p65 inhibition	Reduced infarct, improved recovery, decreased pyroptosis (14)
Enriched Environment	C57BL/6J mice: chronic sleep deprivation	↓ NLRP3/ASC/caspase-1 expression	Direct inhibition of inflammasome assembly	Improved cognition, reduced neuroinflammation (95)
Enriched Environment	Sprague-Dawley rats: diabetes + depression	↓ NLRP3 in hippocampal microglia	PI3K/AKT restoration	Alleviated depressive behavior, neuroprotection, metabolic improvements (94)
Enriched Environment	Sprague–Dawley rats: CUS/depression + fluoxetine	↓ NF-κB activation; shift from M1/iNOS to M2/CD206	NF-κB downregulation → weaker NLRP3 priming	Ameliorated depressive-like phenotype, reduced hippocampal inflammation (113)
Enriched Environment	Sprague–Dawley rats: maternal separation (MS)-induced postpartum depression	↓ NLRP3 in maternal hippocampus	Inhibition of microglial activation and neuroinflammation; upregulated neuroplasticity pathways	Alleviated depressive-like behavior, improved cognition, and promoted synaptic plasticity (93)
Enriched Environment	Sprague–Dawley rats: CUMS/depression + 3-methiladenine (3-MA)	↓ NLRP3 in microglia, ↓ IL-1β, IL-6 and TNF-α	LC3 and Beclin-1 autophagy-mediated inflammation inhibition	Neuroprotective effects on depression and cognitive impairment by inducing autophagy-mediated inflammation inhibition (91)
Physical activity	C57BL/6J mice: MPTP-induced PD model + 10-week aerobic treadmill aerobic exercise	↓ NLRP3 priming and activation; ↓ caspase-1 cleavage and IL-1β/IL-18 releases	Downregulation of NF-κB-dependent priming; suppression of inflammasome assembly; preservation of synaptic integrity	Restored hippocampal neurogenesis, improved cognition, reduced age-related neurodegeneration (114)
Social isolation	C57BL/6J mice: chronic social isolation (8 wk)	↑ hippocampal NLRP3, ASC, caspase-1; ↑ IL-1β/IL-18	NF-κB priming; microglial activation; pyroptosis	Cognitive impairment; synaptic dysfunction (115)
Social isolation	C57BL/6J mice: chronic social isolation + MCC950	↑ NLRP3/caspase-1; altered microglial morphology	Microglial reshaping; canonical NLRP3 signaling	Reversal of cognitive deficits and inflammatory phenotype (116)
Social isolation	Sprague-Dawley rats: depression/AD-like model + fluoxetine	Suppression of TLR4/NLRP3 inflammasome pathway	Activation of Nrf2/HO-1 antioxidant axis and TLR4 inhibition	Reduced brain inflammation, improved behavior, cardio-protection (113)
Social isolation	Sprague-Dawley rats: CUS (21 days) + Shugan Hewei Decoction	↑ NLRP3 expression in serum and cecal mucosa	Gut dysbiosis; peripheral inflammation; epithelial barrier disruption	Reversed anxiety- and depression-like behavior; restored gut barrier (117)

MCAO/R, Middle Cerebral Artery Occlusion and Reperfusion; AD, Alzheimer’s Disease; PD, Parkinson’s disease; CUMS, chronic unpredictable mild stress; MS, maternal separation; 3-MA, 3-methiladenine; MPTP, 1-methyl-4-phenyl-1, 2,3, 6-tetrahydropyridine.

### 4.1 Enriched beyond an ordinary living environment

Environmental enrichment (EE), defined as enhanced sensory, cognitive, and physical stimulation beyond ordinary living conditions, promotes brain function and resilience by promoting neurogenesis, synaptic plasticity, and cognition (118). Preclinical EE paradigms provide larger housing with toys, wheels, tunnels, climbing structures, and social interaction with frequent novelty (89, 119). Compared with standard housing, EE fosters neuroplasticity, dendritic branching, and hippocampal synaptogenesis (89, 90, 120), while also influencing metabolic, endocrine, and immune processes (92, 121, 122). In contrast, impoverished settings with reduced activity and stimulation enhance microglial NLRP3 activation, exacerbating neuroinflammation and pathology (113, 115, 116). Conversely, enriched contexts dampen NLRP3-driven inflammation and improve behavioral and cognitive resilience, highlighting EE as a regulator of inflammasome signaling across conditions (89, 90, 123, 124). Environmental factors regulate NLRP3 activity, by influencing its priming and activation and integrating lifestyle inputs that shape immunometabolic and stress-response pathways (15, 94, 122). Converging preclinical evidence indicates that EE attenuates NLRP3 signaling and downstream inflammation across distinct pathophysiological contexts (**Table 2**).

In cerebral ischemia/reperfusion, EE improved recovery and reduced infarct size by suppressing NLRP1/NLRP3, caspase-1 activation, GSDMD-mediated pyroptosis and IL-1β/IL-18 releases via NF-κB inhibition compared to standard housing (14). In chronic sleep deprivation, EE rescued cognition and downregulated hippocampal NLRP3/ASC/caspase-1 levels, limiting inflammasome assembly (95). In diabetes with comorbid depression, EE alleviated depressive-like behavior, improved glucose regulation, reduced neuronal apoptosis, and suppressed microglial NLRP3 alongside PI3K/AKT pathway restoration, a negative regulator of inflammasome activation (94). More broadly, under stress- and depression-related paradigms, EE reprogramed microglia toward anti-inflammatory phenotypes (15) and restrained NF-κB signaling, reducing NLRP3 priming (91, 93). Beyond EE, physical activity exerts potent strong anti-inflammatory and immunomodulatory effects mediated by NLRP3 inflammasome. In middle-aged mice, Zhao et al. (2025) demonstrated that aerobic exercise restored hippocampal neurogenesis, improved cognition, and counteracted age-related neurodegeneration by reducing NLRP3 priming and activation, thereby preserving synaptic integrity and attenuating neuroinflammation (114).

### 4.2 Social isolation as an adverse living condition

Social connectedness supports health, whereas reduced interaction impairs cognition and behavior (125). In rodents, social isolation models psychosocial stress, driving microglial activation and NLRP3 inflammasome–mediated neuroimmune dysregulation (**Table 2**). Chronic social isolation increases hippocampal levels of ASC, caspase-1, and IL-1β/IL-18, driving cognitive deficits, depressive-like behavior, and impaired synaptic plasticity. Niu et al. (2020) further showed that isolated mice exhibited marked hippocampal upregulation of inflammasome proteins and cytokines without peripheral changes, underscoring a CNS-specific mechanism. Both the antibiotic minocycline and the selective inflammasome inhibitor MCC950 reversed NLRP3 activation and rescued cognition (115). Li et al. (2021) showed also that MCC950 prevented social isolation-induced depressive-like behavior, normalized microglial morphology and restored NLRP3/caspase-1 signaling in male mice, indicating that inflammasome blockade promotes behavioral resilience (116). Extending these findings, Abu-Elfotuh et al. (2022) demonstrated that the antidepressant fluoxetine counteracted isolation-exacerbated brain and cardiovascular pathology in an AD-like depression model by activating Nrf2/HO-1, an antioxidant pathway that limits ROS accumulation and thereby inhibits NLRP3 signaling (113). In contrast to CNS-focused studies, Yue et al. (2021) investigated the peripheral effects of chronic psychosocial stress using a chronic unpredictable stress (CUS) model that included social isolation as a key stressor (117). They showed their contribution to NLRP3-driven peripheral inflammation and highlighted the gut-brain axis as a parallel mediator of stress-related behavioral outcomes. Male rats exposed to 21 days of CUS developed depressive- and anxiety-like behaviors, weight loss, and gut alterations, with a significant upregulation of NLRP3 in both serum and cecal mucosa. Treatment with *Shugan Hewei Decoction*, a traditional multi-herbal remedy, reversed behavioral deficits, improved gut barrier function, and suppressed peripheral inflammasome activation (117).

## 5 Conclusion

The NLRP3 inflammasome is emerging as a central player in brain disorders and systemic conditions with neurological repercussions, underscoring its relevance within the CNS and potential as a biomarker (126). The pathway toward precision medicine will require sex-informed approaches to NLRP3 research, spanning preclinical studies and therapeutic development, rather than relying on uniform models that risk overlooking meaningful differences. While sex-dependent regulation of NLRP3 has been explored in some immune-related brain conditions, comparable investigations in mental health disorders remain limited. This gap is particularly pressing, as NLRP3 influences cognition and behavior and may contribute to sex disparities in neurocognitive and neurobehavioral disorders. Importantly, critical developmental windows such as puberty and aging act as “reset points” for inflammatory set-points in a sex-dependent manner. Fluctuating ovarian steroids (puberty, perimenopause, menopause) and androgens (puberty, aging) shape microglial phenotype and NLRP3 sensitivity, as shown in four-core genotype paradigms disentangling chromosomal and gonadal contributions (127, 128). These dynamics highlight the need for studies stratified by pubertal stage and aging stage to fully map sex-by-age interactions on NLRP3 priming and activation in CNS cells.

Concurrently, multiple environmental exposures profoundly modulate NLRP3 activity. Its heightened sensitivity to adverse inputs can drive maladaptive outcomes, including depression-like behaviors, cognitive decline, and stress vulnerability. Yet this sensitivity also offers an opportunity for lifestyle and environmental interventions, alongside pharmacological strategies, to dampen inflammation and preserve brain health, an avenue still underexplored. Moreover, biological sex, long considered static, is now recognized as dynamic in constant interplay with the exposome, with implications for sex-specific health outcomes and NLRP3-mediated mechanisms that can no longer be overlooked (129).

Given the dual influence of intrinsic (sex) and extrinsic (environment) factors on NLRP3, we suggest that a deeper understanding of their interplay, with NLRP3 as a central hub, could foster a more personalized and effective medicine, in which targeted non-pharmacological strategies complement pharmacological treatments (130). Such integration may overcome limitations of exclusive pharmacological NLRP3 inhibition, risks diverting inflammation toward alternative inflammasome pathways with harmful consequences (131–133). Addressing the complex interplay between intrinsic and extrinsic determinants of NLRP3-driven neuroimmune responses may ultimately enable more precise prevention, improved therapeutic efficacy, and better long-term neurological outcomes.
